# Patient insights on living with idiopathic inflammatory myopathy and the limitations of disease activity measurement methods – a qualitative study

**DOI:** 10.1186/s41927-020-00146-3

**Published:** 2020-09-21

**Authors:** Alexander Oldroyd, William Dixon, Hector Chinoy, Kelly Howells

**Affiliations:** 1 NIHR Manchester Biomedical Research Centre, Manchester University NHS Foundation Trust, Manchester Academic Health Science Centre, Manchester, UK; 2 Centre for Musculoskeletal Research, University of Manchester, Manchester Academic Health Science Centre, Manchester, UK; 3 Centre for Epidemiology Versus Arthritis, University of Manchester, Stopford Building, Oxford Road, Manchester, M13 9PT UK; 4 Department of Rheumatology, Salford Royal NHS Foundation Trust, Salford, UK; 5 NIHR Greater Manchester Patient Safety Translational Research Centre, University of Manchester, Manchester, UK; 6 Centre for Primary Care and Health Services Research, University of Manchester, Manchester, UK

**Keywords:** Myositis, Qualitative, Outcome assessment, Pain, Fatigue

## Abstract

**Background:**

The idiopathic inflammatory myopathies (IIMs) are chronic autoimmune conditions, typically resulting in proximal muscle weakness and impacting upon quality of life. Accurate measurement of IIM disease activity is imperative for appropriate medical management and carrying out valid clinical trials. The International Myositis Assessment and Clinical Studies Group (IMACS) “Disease Activity Core Set Measures” are the current gold-standard of IIM disease activity assessment. Anecdotally, patients with an IIM report that the IMACS Core Set Measures and other available methods do not necessarily capture their perceived disease activity. Investigating the patient experiences of living with an IIM and their views on the accuracy of the IMACS Core Set Measures will provide valuable insights for both clinical and research purposes.

**Methods:**

Eighteen interviews with patients with an IIM were carried out and analysed thematically, using a grounded theory approach. Experiences on living with an IIM and perceptions on the accuracy of disease activity measurement methods were explored.

**Results:**

Interview analysis revealed four themes: 1) fatigue, 2) pain, 3) day-to-day symptom variation, 4) limitations of creatine kinase levels and manual muscle testing.

**Conclusions:**

This study has provided valuable insights into patient experiences of living with an IIM. Aspects of IIM disease activity perceived not to be wholly measured by the IMACS Core Set Measures have also been identified. These findings have implications for future IIM clinical care and research, in particular providing justification for research into pain, fatigue and symptom variation.

## Background

The idiopathic inflammatory myopathies (IIMs) are a group of chronic autoimmune conditions that can lead to widespread inflammation and damage [1, 2]. A number of clinical IIM subtypes are recognised, including dermatomyositis (DM), juvenile DM (JDM), polymyositis (PM), immune-mediated necrotizing myopathy (IMNM), anti-synthetase syndrome (ASS), and sporadic inclusion body myositis. A wide variety of IIM manifestations can occur, including muscle inflammation (myositis) of proximal limb muscles leading to weakness [3–6], skin inflammation, interstitial lung disease and an increased malignancy risk. The disease course is variable, with many patients reporting unpredictable episodic exacerbations of symptoms and disability [7].

Living with IIMs can impact significantly on quality of life [7–9]; this impact on quality of life being a combination of disease manifestations, requirement of repeated medical interactions and treatment complications.

Extensive qualitative research by OMERACT has investigated the impact of living with IIM [10–13]. Focus groups, one-on-one interviews and a Delphi survey identified a number of themes, which included 1) predominance of pain and fatigue, 2) the emotional consequence of the disease, 3) symptom variability, 4) limitations in participation in society, 5) impact of relationships with healthcare providers, 6) insomnia and 7) cognitive dysfunction. Identification of these themes has informed subsequent research and development of IIM-specific outcome measurements, such as the Myositis Activities Profile [14] and the Functional Index [15].

“Disease activity” is defined as the features of a disease that are potentially reversible with treatment, such as active myositis, whereas “disease damage” refers to permanent and irreversible features that are a consequence of disease activity, such as muscle fibrosis [16]. Accurate assessment of disease activity is imperative to allow for appropriate medical management. A number of valid measurements of myositis disease activity have been combined into the International Myositis Assessment and Clinical Studies Group (IMACS) “Disease Activity Core Set Measures” [17], which is currently used as the gold-standard of IIM disease activity assessment. The IMACS Disease Activity Core Set Measures include manual muscle strength testing (MMT), blood tests for creatine kinase (CK) levels, the Health Assessment Questionnaire (HAQ) (a validated measure of functional ability), and both patient and physician “global assessment” of disease activity. These measurements can be used in both clinical and research settings when disease activity quantification is required.

Anecdotally, many people with an IIM report that the IMACS Disease Activity Core Set Measures, and other clinical methods, such as MRI scanning, do not necessarily capture their own perception of disease activity. This discrepancy between clinical measurement and patient-perceived disease activity may result in an incomplete quantification of disease activity, thus limiting and misfocusing clinical interventions otherwise aimed to improve symptoms, quality of life and function.

Investigation of the patient-perceived accuracy of IIM disease activity assessment methods may provide insights that will inform future development of new outcome measurements or the tailoring of existing methods. Also, further understanding of the patient-experience can assist health care professionals to better comprehend the impact of living with IIM.

In this study, we conducted qualitative interviews to understand the patient experience of living with IIM and their perceptions of the ability of currently available methods to measure IIM disease activity accurately.

## Methods

Recruitment and qualitative interviews were carried out as part of the Myositis Physical Activity Device (MyoPAD) study. The study aimed to design and trial the MyoPAD system, which entails a smartphone-based app, allowing entry of daily PROMs, and a thigh worn accelerometer sensor, which allows for remote and continuous characterisation of gait parameters. Results of quantitative analysis of data collected through the MyoPAD study will be published separately. This paper will report the results of analysis of qualitative interview data, collected at the time of recruitment.

Participants were recruited from the specialist neuromuscular clinic at Salford Royal Hospital, Salford, UK. Participants were invited to join the MyoPAD study if they were aged 18 years or over, had a physician-verified IIM diagnosis (International Myositis Classification Criteria Project [18] or European Neuromuscular Centre [19] criteria) of PM, DM, IMNM or ASS, owned their own smartphone (Apple or Android; to allow daily app data entry) and had regular access to their own Wi-Fi connection (to allow frequent data transfer). Participants unable to enter data via an app or walk independently were excluded from recruitment. Participants unable to converse in English were also excluded as the study did not have capacity to conduct interviews in another language.

All participants were invited to interviews (maximum 1 hour in duration) at their time of recruitment to the MyoPAD Study. Participants received an information sheet prior to the interview with a list of possible areas of discussion. A.O. conducted all interviews, which took place in Salford Royal Hospital’s Clinical Research Facility. Participants were given the option to attend the interview with a partner or friend who were also invited to contribute to the discussion where appropriate following verbal consent from the participant. Interviews followed a semi-structured format with pre-prepared interview guides (see Supplementary Material for interview topic guide). Interviews were audio-recorded and transcribed.

During the interview, participants were invited to discuss the following: their experiences of living with an IIM, symptoms, symptom variability, perceived ability to convey symptoms during a clinical consultation, and views on current methods of IIM disease activity measurement. Participants were also invited to discuss aspects of living with an IIM that they perceived to be under-recognised or not fully assessed in clinical consultations.

Interview transcript data was analysed thematically, using a grounded theory approach [20]. Coding was carried out by A.O. and K.H. using NVivo qualitative data analysis software (QSR International Pty Ltd., Version 11, 2015). Initial coding formed the basis for long descriptive accounts of the coded data that were circulated, discussed and refined during analysis meetings between A.O. and K.H. Initial codes were grouped to form core thematic categories based on multiple sources of interview data.

The Greater Manchester Central Research Ethics Committee approved the study (ref. 18/NW/0676). Informed written consent was provided by all study participants prior to interviews. Consent included permission to record interviews and reproduce anonymised quotations.

## Results

Eighteen (61% female) participants with a verified IIM diagnosis took part in the interviews. Four participants were accompanied by a friend or partner; only one partner quotation contributed to theme formation. The median age of the cohort was 52 years (IQR 44, 56) with a median IIM disease duration of 5 years (IQR 2, 6). Analysis of baseline interview data revealed four main themes. Each theme will be discussed in turn, with accompanying quotations (Table 1).

**Table 1:** Quotations from participants

Quotation number	Quotation
**Fatigue**	
1	*“I would say that the fatigue has more of an effect than the pain. Sort of, I get aches but that’s not the worst but, it’s just the sort of real exhaustion that is the worst.”*
2	*“Fatigue is my main component. I think for me rather than muscle weakness and rather than pain it’s fatigue, concentration and focus.”*
3	“So washing my hair is not too bad and then when I’m coming to dry my hair, that can be difficult, holding my hands up.”
4	*“What I’ve been able to maintain is like, short, sharp things. So if I have to do something quite quick, it doesn’t bother me.”*
5	*“My daughter likes her in plaits, and trying to plait her hair, that can get quite tiring, I have to rest my arms.”*
6	*“Then you start to do daily chores like having a shower or, in my case because I’ve got a little girl; making breakfast for her, helping her to get dressed, ironing her uniform for school. All of those day to day things that you used to do without thinking about it; adjusting the shower head, washing your hair.”*
7	*“I know tomorrow is going to be a rest day because of the drive today.”*
8	*“Profound weakness with my myositis seems to be much more background.”*
9	*“I think the fatigue side seems to be missed quite a bit, that’s never sort of talked about too much, it’s more just* “*what’s your strength” and “are you breathing okay”.”*
10	*P12’s partner: “The logistics. Where is the toilet going to be? You know, where are the steps? You know, we have to plan everything. Everything [PARTICIPANT] does needs careful planning, because it could be detrimental to her wellbeing.”*
**Pain**	
11	*“It’s not pain, it’s a really hard one to describe and I know other people will have said the same thing to you, it’s not pain for me it’s a muscle burning that I get. And I wouldn’t describe it as painful, I’d describe it as uncomfortable but yeah you can feel something going on but it’s really hard to explain and burning is the nearest I can get to it.”*
12	*“I get, what I call proper pain and then, in my legs and arms if I lift them or try to lift anything or try to hold them up for a length of time, washing my hair, that kind of thing, then I’ll feel like a burn like you get with extreme exercise.”*
13	*“I don’t feel that anyone [CLINICIANS] I’ve spoken to recognise pain as part of it.”*
**Day-to-day symptom variation – characterisation of good and bad days**	
14	“*I will be fine one day, the next day I can feel absolutely terrible.”*
15	*“I could be tired for an hour and be fine the next hour, particularly in the early days.”*
16	*“There’s a hill that leads up home, and if I’m having what I call a good day I can charge up it, and then a day where I know is a flare, I’ll stop two or three times.”*
17	*“But I just feel like I’ve been hit by a bus. And you just ache. But the next day everything’s okay.”*
18	*“I suffer greatly in the mornings, first thing. I can tell by the way I am first thing in the morning, I tend to get a grasp of how the day is going to go.”*
19	*“You do tend to do far too much on the good days, and then you pay for it a couple of days later.”*
20	*“There’s definitely a finite resource with the fatigue effect and I know that’s very difficult to quantify.”*
21	*“My condition is up and down all over the place so it can almost change day on day, which is ridiculous. Family and friends, that’s the hardest thing they struggle to get their heads around. I will be fine one day, the next day I can feel absolutely terrible.”*
22	*“I think doctors can’t understand either, that some days you can be quite well and other days you can be really, really bad.”*
23	“*On the outside we look normal, we look well, you know, everybody says, “oh you look really well” … actually if they knew what a struggle it was for me to actually get to be somewhere … people don’t recognise the exhaustion and the tiredness that can go with it and the effort you have to put into doing the simplest tasks.”*
24	*“Myositis isn’t a common condition, so people don’t really understand what it is about so, therefore they’re probably more reluctant to ask what that is… So, it’s unknown isn’t it. Therefore, people tend to respond to unknown things with oh yeah, yeah, you’re looking okay, that’s fine it must be difficult, but I don’t think they quite know.”*
25	*“A lot of people have said that they’ve come out of the doctor’s office feeling quite frustrated because they haven’t been able to convey to the doctor that they feel the way they do.”*
26	*“Because I could have a really bad week in the first month after seeing the rheumatologist and then, by the time I’ve got there, I’m quite dapper and, you know, you walk in but, there’s been them days where you are crying in pain or you just feel so fatigued and brain fogged that…so, yeah because it’s so variable you can look totally different than you have been.”*
27	*“Your brain tends to remember good days...unfortunately, your brain goes back to a healthy state quite quickly.”*
28	*“So, I actually wrote it down in the book, sort of, what it was that started and when and how that had gone. So, I could go in and say this is what’s happened because I knew I wouldn’t remember what to say, this is how it was.”*
29	*“I get the CK checked once every few months, but that never correlates to having a flare.”*
**Limitations of CK levels and MMT as measurements of disease activity**	
30	*“My own feelings are that I quite often feel worse than the results that come back from any of the tests [CK level] really.”*
31	*“Although there is a three hundred limit for normal, when my CK score goes from about one-sixty, one-seventy to two-forty, two-fifty, it is still within the normal range but I am in full flare.”*
32	*“I feel that because my CK levels have come down that I just feel that’s what people are happy with and because it’s all judged on that.”*
33	*“I find medical professionals and different people interpret it [CK levels] in different ways.”*
34	*“I could walk a short distance, but if I had to keep walking then I would really struggle and probably need to go to sleep afterwards. Like lifting an arm up, I can do it once but if I had to hold the arm up for any length of time, I wouldn’t be able to do it.”*
35	*“I don’t think it gives a very accurate representation of strength because I think people try really hard to resist and showing how strong they can be, because that’s your nature isn’t it, you want to try and do well in it, but actually the effort that’s involved can really exhaust you, and as much as anything it’s repeating those movements.”*
36	*“I think the fatigue side seems to be missed quite a bit, that’s never sort of talked about too much, it’s more just is what’s your strength and off you go.”*

*CK* creatine kinase, *MMT* manual muscle testing, *IIM* idiopathic inflammatory myopathy

### Fatigue

Fatigue, as a manifestation of reduced physical endurance, was the most common and prominent symptom, and was reported by all participants (Textbox quotations 1 and 2). Activities of daily living (ADLs) were frequently reported to be affected by fatigue. In particular, ADLs that require sustained shoulder abduction, such as hair drying/styling and telephone use, were frequently reported to be affected (Textbox quotation 3). In contrast, participants reported less impact upon shorter duration tasks, such as dressing; some also reported tailoring their ADLs to purposefully include such short duration needs of exertion (Textbox quotation 4). The impact of fatigue upon their ability to wash, dress or care for children was a source of great concern for a number of participants (Textbox quotation 5).

A number of participants reported instigating strategies to help cope with or ameliorate their fatigue, which include planning “rest days” (Textbox quotations 6 and 7).

Fatigue was reported to be a more important symptom compared to perceived muscle weakness, which was not associated with perceived variations of disease activity or a factor that directly impacts upon quality of life (Textbox quotations 2 and 8). Multiple participants explained that fatigue is not commonly assessed or addressed during clinical consultations. A reduced awareness amongst clinicians of fatigue as an IIM-related symptom was suggested as a possible reason for this omission (Textbox quotation 9).

Travel was reported to be a particularly fatigue-inducing activity, with a number of participants reporting limiting their travel to only the essential. Also, the need to plan all aspects of travel details, such as the presence of steps or slopes, the location of rest facilities and the assurance that an emergency contact be available, was a source of concern and sense of limitation to a number of participants, their partners and families (Textbox quotation 10). This need to meticulously plan all travel, even short distances, appeared to add to a sense of loss of independence and their condition dominating many aspects of their life.

### Pain

Second to fatigue, pain was a key symptom. The character of pain was reported in a number of different ways, including “spasm”, “muscle burning”, “feeling like you’ve run a marathon”, and so severe that “it does bring you to tears”, however it was generally reported that conveying the nature of the pain was difficult (Textbox quotation 11).

Pain was noted to be linked to carrying out physical activities, even of low intensity. The occurrence of pain whilst carrying out daily activities was a particularly troublesome symptom (Textbox quotation 12). A number of participants acknowledged that pain may only be experienced when they exceed a certain level of duration of physical exertion, typically associated with certain ADLs. Participants reported needing to therefore be more purposeful and deliberate in planning and executing ADLs, choosing which were essential and which can be postponed until they felt capable.

### Day-to-day symptom variation – characterisation of good and bad days

Symptom variation was reported by the majority of participants. Many participants reported that symptoms, particularly fatigue and pain, varied on a day-to-day or even hour-to-hour basis (Textbox quotations 14 and 15). Many participants recognised the occurrence of “good” and “bad” days, however these varied between participants and impacted upon their function in individual ways, depending on their lifestyle. Good days were characterised by increased physical stamina, thus allowing increased fulfilment of activities of daily living (ADLs), improved walking ability, a perception of higher energy levels and diminished or absent pain (Textbox quotation 16). In contrast, “bad days” were characterised by higher levels of pain and fatigue and the presence of malaise, resulting in difficulty carrying out ADLs (Textbox quotation 17). Participants commonly noted an ability to identify if they were going to experience a bad day according to their symptoms at the time of waking (Textbox quotation 18). A number of participants described more severe symptoms, such as pain and fatigue, in the days following a “good day” and suggested that this may be due to over-exertion (Textbox quotation 19).

Energy rationing, i.e. conserving one’s own energy by carrying out only certain physical activities, was commonly reported, especially by participants with longer IIM disease durations, as a coping strategy to prevent debilitating fatigue associated with over-exertion. A number of participants expressed that only a certain amount of energy was available to them each day, which, if exceeded, would result in subsequent “bad” days characterised by worsening of symptoms including fatigue and pain. Further, the difficulty in quantifying this amount of energy was acknowledged (Textbox quotation 20). The finite amount of energy perceived to be available for a certain day was described as a “sugar cube” by one participant (P4), with them “constantly trying to offset” against physical exertion.

Symptom fluctuation was reported to be under-recognised by both family members/friends and clinicians (Textbox quotations 21 and 22). A number of participants explained that this non-recognition of symptoms and symptom variation may be due to the absence of clear visual indicators of illness or symptoms. This lead to a perception of IIM being an “invisible disease” (Textbox quotation 23). Participants explained the ensuing underlying frustration that non-recognition of IIM can cause, in part due to low levels of public awareness and rarity of the condition (Textbox quotation 24).

The combination of symptom variation, infrequent clinical review and perception of difficulty conveying the wide variety of symptoms was a source of concern reported by many participants (Textbox quotation 25). In particular, a number of participants recognised that the assessment at a clinic appointment may not capture fluctuations of disease activity since the previous assessment (Textbox quotation 26). A further reported limitation of infrequent clinical reviews is the difficulty to recollect, perhaps multiple symptom fluctuations that may have occurred months previously, with “good days” and improved symptoms being preferentially recollected, therefore potentially limiting the clinician’s understanding of the patient experience (Textbox quotation 27). A small number of participants reported keeping a diary of symptoms, flare occurrence and perceived causes, thus improving symptom variation recollection at the time of appointment (Textbox quotation 28).

### Limitations of CK levels and MMT as measurements of disease activity

Many participants provided detailed views on their perception of the ability of CK levels and MMT to assess IIM disease activity. The majority of participants explained their experiences of changes of CK levels not corresponding to symptom fluctuations (Textbox quotation 29). Even the small number of participants who felt that the CK was associated with their disease activity felt that it underestimated the extent of their symptoms (Textbox quotation 30). The comparison of an individual CK level against the normal laboratory range was criticised, with participants perceiving the comparison to their own baseline perhaps being more appropriate (Textbox quotation 31). Further, a number of participants perceived that clinicians may base their assessment of IIM disease activity solely on the CK level, without taking extra-muscular manifestations into account (Textbox quotation 32). A number of participants also suggested that changes of the CK level is interpreted differently by various clinicians (Textbox quotation 33).

Drawbacks of MMT, as a measure of disease activity, were also reported by many participants, these included differing results between clinicians, the inability of MMT to assess fatigue on sustained muscle use and inability to capture day-to-day strength variation (Textbox quotation 34). Participants also noted the inability of MMT to assess endurance, which, as described earlier, is a major source of functional limitation. It was also reported that the MMT assessment may vary greatly depending on other factors, in particular patient motivation and a clinician’s prior knowledge of weakness (Textbox quotation 35). A perception of clinicians relying predominantly on the MMT to assess disease activity was conveyed, with participants feeling that hidden symptoms, such as fatigue, would be missed, as described earlier (Textbox quotation 36).

## Discussion

Our study aimed to understand the patient-reported experience of living with IIM and to explore patient perceptions of the ability of currently available methods to accurately measure disease activity. This qualitative study has provided a number of valuable insights.

Fatigue and pain were reported to be predominant symptoms, resulting in reduced function and impacting upon quality of life. Our findings replicate findings reported in a number of OMERACT studies, which, as mentioned earlier, investigated patient experiences of living with IIM and identified that both pain and fatigue were predominant symptoms. Of the small number of studies that have quantitatively measured fatigue and myalgia (muscle-related pain) in IIM populations, scores were typically high (mean 7/10 for fatigue and 4/10 for pain in DM) [21].

In combination with infrequent clinical reviews and perceived drawbacks of MMT and CK levels, participants felt that disease activity could not be wholly quantified by their clinicians. A number of studies have investigated the validity of MMT assessment and interpretation of CK levels. Both MMT and CK levels have been deemed to accurately represent IIM disease activity and therefore appropriately form two of the six IMACS Disease Activity Core Set Measures [17]. Rider et al reported high levels of convergent construct validity, internal reliability and inter-rater reliability [22]. In this study, disease activity was represented by physician global activity score, function (HAQ) and MRI changes, however fatigue and pain were not included. Development and validation of quantitative IIM-specific measurements of fatigue, pain and other symptom qualities, such as day-to-day variation, in the form of patient reported outcome measures could potentially enhance IIM disease activity assessment. OMERACT have recently recommended inclusion of pain and fatigue in the core set for IIM clinical trial outcome measures (“life impact area”) and measurement instrument selection will be carried out in the near future [23]. This recognition of the importance of patient symptoms follows changes in 2014 for rheumatoid arthritis clinical trials when OMERACT added in fatigue to the Core Domain Set following patient participant focus group input [24, 25]. Participants reported that fatigue results in difficulty carrying out particular ADLs, such as combing hair or sustained telephone use. However participants perceived that such difficulties are not clearly captured or quantified in routine clinical practice. Although validated measurements of function, such as the HAQ [26] or SF-36 [27], assess such activities (e.g. ability to “wash and dry your body” – HAQ), these responses are assimilated into an overall conglomerated score, thus potentially falsely missing a patient’s limited ability to complete such a task. Perhaps IIM-specific outcome tools, such as the Adult Myopathy Assessment Tool [28] or Functional Index [15], that measure task endurance could more accurately quantify functional limitation of ADLs.

Day-to-day symptom variation was reported by the majority of participants; this being in contrast to the traditional understanding of IIM-related symptoms being less variable. Regardt et al reported that symptom variation is an important facet of the IIMs [29]. This was in association with cognitive dysfunction, limitations in daily activities and participation in society. To our knowledge, no other study has investigated the detailed variations of IIM-related symptoms in either a qualitative or quantitative manner. It is imperative that, during a consultation, clinicians caring for people with IIM are able to understand variations of symptoms and their impact on quality of life. Further, infrequent clinical assessment, alongside frequent symptom variation, may risk inaccurate quantification of disease activity assessment. Inaccurate or selective recall may also impact consultations and comprehensive conveyance of symptoms, as described by our participants and reported in previous studies [30, 31]. Novel methods, such as collection of daily patient-reported outcome measures, may provide a solution and allow quantification of symptom variation, for use in both clinical and research settings. Recent development of a smartphone-based method of daily symptom tracking in rheumatoid arthritis has demonstrated added patient benefit, enhancing clinical consultations and improving symptom variability recognition [32]; development of a similar method, tailored to the IIMs, could potentially remedy a number of limitations described by the participants of our study.

Finally, IIMs were felt to be an “invisible” set of diseases, with limited understanding from friends, colleagues and clinicians being a source of concern and social isolation. The concept of a hidden disease is present in other chronic conditions, such as rheumatoid arthritis [33], systemic lupus erythematosus [34] and fibromyalgia [35]. Difficulty in communicating symptoms to clinicians, friends, family members and colleagues is common in other chronic diseases that lack outward visibility [36, 37]. Both the hidden component of IIM and low awareness amongst the general and healthcare populations may further add to communication difficulties in social, professional and medical conversations. Again, novel continuous remote monitoring methods may facilitate reporting of hidden disease aspects and enhance clinicians’ understanding. Impact of IIM upon “participation in society” was also reported from focus group work carried out by Regardt et al. [29] and Chung et al reported higher levels of social isolation, compared to rheumatoid arthritis, spinal osteoporosis and knee osteoarthritis [38]. Social isolation and a perception of lack of understanding from others is commonly reported in other disabling chronic conditions, such as rheumatoid arthritis [39, 40], Parkinson’s Disease [41] and multiple sclerosis [42].

Strengths include the novelty of carrying out a detailed qualitative study in an IIM population with a particular focus upon participant-perceived accuracy of disease activity assessment methods. Another strength includes the fact that the demographics of the population mirror that of the general adult IIM population, thus potentially aiding generalisability, however this may be mitigated by recruitment bias, which is the most important potential limitation of this study. Although appropriate for the MyoPAD study, the exclusion criteria will have precluded certain sub-groups, such as those with more profound walking disability, whose experiences of living with an IIM may differ from our recruited cohort. Self-selection may also have occurred, with only particularly motivated patients agreeing to take part in one-to-one interviews, thus potentially excluding certain sub-groups. This potential recruitment bias may affect generalisability of findings, therefore validation of results in other IIM populations is warranted. Future qualitative research that includes a cohort with varying disease activity, disease duration and additional IIM subgroups broader cultural backgrounds is warranted and will provide a wider perspective on this topic.

Our findings have the potential to influence clinical practice in a number of ways. Clinicians should be mindful to assess regularly and quantify, where possible, pain and fatigue levels when reviewing IIM patients. Regular assessment of such symptoms with a focus on resolution as a treatment aim may influence self-management and positively impact patient quality of life. Clinicians should also be aware of frequent symptom variability and ensure enquiry into variation since the last assessment.

The predominance of pain and fatigue as major perceived symptoms of IIM are an important and key finding. Reporting of frequent symptom variability is also illustrated within our study. The model of conventional pre-planned and infrequent clinic appointments may impact on accurate disease activity assessment, and thus may not be helpful for our patients. A future research agenda into novel methods to address these issues could greatly enhance patient care. Other suggested research includes identifying if reported symptoms, including pain and fatigue, are due to IIM disease activity and/or damage; such distinction will allow for focused development of corresponding outcome measures.

## Conclusions

In conclusion, our study has provided in depth qualitative insights into the patient-experience of living with IIM, highlighting that pain and fatigue are predominant symptoms, alongside frequent symptom variation and the impact of current methods of IIM disease activity assessment. Consideration should now be given to capturing pain and fatigue along with other routine clinical assessments in a more frequent manner for IIM patients.

## Supplementary information

**Additional file 1.**

## Data Availability

The datasets used and/or analysed during the current study available from the corresponding author on reasonable request.

## Abbreviations

ASS: Anti-synthetase syndrome
ADL: Activities of daily living
DM: Dermatomyositis
HAQ: Health Assessment Questionnaire
IIM: Idiopathic inflammatory myopathy
IMACS: International Myositis Assessment and Clinical Studies Group
IMNM: Immune-mediated necrotizing myopathy
JDM: Juvenile dermatomyositis
MMT: Manual muscle testing
MyoPAD Study: Myositis Physical Activity Device Study
OMERACT: Outcome Measures in Rheumatology
PM: Polymyositis
SF-36: 36 Item Short Form Survey
