# Supplementary material for: Patient insights on living with idiopathic inflammatory myopathy and the limitations of disease activity measurement methods – a qualitative study
Source: BMC Rheumatol. 2020 Sep 21;4:47. doi: 10.1186/s41927-020-00146-3 (PMC7504651; doi:10.1186/s41927-020-00146-3)
Supplement: Supplementary file 1 — Additional file 1. [file 41927_2020_146_MOESM1_ESM.docx]

**Interview topic guide: patient participant baseline interview**

**Study Title: The Myositis Physical Activity Device (MyoPAD) Study**

**Views on current methods of IIM disease activity measurement:**

- Do you think current methods, such as blood tests and MRI scans, measure your disease activity accurately?
- What aspects of myositis disease activity are measured well by current methods?
- What aspects of myositis disease activity are measured poorly by current methods?
- Do you think the frequency of assessment impacts the detection of changes of disease activity?

**Views and opinions on relationship between IIM (referred to as “myositis” throughout) disease activity and physical activity:**

- Do you think your myositis and physical activity capability are linked?
- What particular physical activities do you think myositis affects?
- Do you think your walking pattern is affected by myositis?
- Do you think your walking speed is affected by myositis?
- Does reduction in physical activity from your myositis affect your day-to-day activities?
- Has this affected your social life, relationships with family, friends or work colleagues?
- Do you think myositis affects your mood?
- Do you think your physical activity capability is related to treatment for your myositis?
- Do you think you can tell when your myositis is getting better or getting worse?

**Views and opinions on using a smartphone app to collect data and the potential value:**

- What are your views about storing and sharing health data collected by monitoring symptoms etc by smartphone apps?
- Do you have any concerns about this method?
- What do you think are the benefits of this type of monitoring?
- Do you think the MyoPAD app will affect how you manage your condition/health?
- Do you think the MyoPAD app will affect how clinicians manage your condition/health?

**Previous experience of monitoring symptoms and activity, and use of health apps:**

- Have you done any previous monitoring of symptoms and physical activity? If so, can you give examples?
- Have you any experience of using other health related apps? If so, can you give examples and describe how you found using them?

The interviewer will give a University of Manchester smartphone to the interviewee. The smartphone will have a prototype version of the MyoPAD app installed.

The interviewer will also give a SENS Motion Plus accelerometer patch to the interviewee.

**First impressions and expectations of using the MyoPAD app and SENS Motion Plus accelerometer patch:**

- How do you think you will find using the MyoPAD app?
- Do you think you will be happy to wear the patch?
- Do you anticipate any problems using the MyoPAD app and patch?

**Views about symptom monitoring using the MyoPAD app:**

- What are your first impressions of the app?
- Do you think you will you be happy to complete the app’s questions every day?
- Do you think you will be happy to complete the app’s questions every day for a 90 day period?
- Are there any other questions that you would like to see in the app?

**Changes to app:**

- Are there any other aspects of myositis that you would like to be assessed by the MyoPAD app?
- Are there any alterations to the app’s interface that you could suggest?

**Any other issues you would like to raise?**
